# AMPK, a metabolic sensor, is involved in isoeugenol-induced glucose uptake in muscle cells

**DOI:** 10.1530/JOE-15-0302

**Published:** 2016-02

**Authors:** Nami Kim, Jung Ok Lee, Hye Jeong Lee, Yong Woo Lee, Hyung Ip Kim, Su Jin Kim, Sun Hwa Park, Chul Su Lee, Sun Woo Ryoo, Geum-Sook Hwang, Hyeon Soo Kim

**Affiliations:** 1 Department of Anatomy, Korea University College of Medicine, Ewha Womans University, Seoul, 136-701, South Korea; 2 Department of Medicine, Korea University College of Medicine, Seoul, South Korea; 3 Integrated Metabolomics Research Group, Korea Basic Science Institute (KBSI), Western Seoul Center, Seoul, South Korea; 4 Department of Life Science, Ewha Womans University, Seoul, South Korea

**Keywords:** AMPK, isoeugenol, glucose uptake, p38 MAPK

## Abstract

Isoeugenol exerts various beneficial effects on human health. However, the mechanisms underlying these effects are poorly understood. In this study, we observed that isoeugenol activated AMP-activated protein kinase (AMPK) and increased glucose uptake in rat L6 myotubes. Isoeugenol-induced increase in intracellular calcium concentration and glucose uptake was inhibited by STO-609, an inhibitor of calcium/calmodulin-dependent protein kinase kinase (CaMKK). Isoeugenol also increased the phosphorylation of protein kinase C-α (PKCα). Chelation of calcium with BAPTA-AM blocked isoeugenol-induced AMPK phosphorylation and glucose uptake. Isoeugenol stimulated p38MAPK phosphorylation that was inhibited after pretreatment with compound C, an AMPK inhibitor. Isoeugenol also increased glucose transporter type 4 (GLUT4) expression and its translocation to the plasma membrane. GLUT4 translocation was not observed after the inhibition of AMPK and CaMKK. In addition, isoeugenol activated the Akt substrate 160 (AS160) pathway, which is downstream of the p38MAPK pathway. Knockdown of the gene encoding AS160 inhibited isoeugenol-induced glucose uptake. Together, these results indicate that isoeugenol exerts beneficial health effects by activating the AMPK/p38MAPK/AS160 pathways in skeletal muscle.

## Introduction

In the muscles, glucose uptake occurs through two major pathways, namely insulin-dependent glucose uptake and non-insulin-dependent glucose uptake. In muscle cells, binding of insulin to insulin receptors increases the activation of the phosphatidylinositol-3-kinases (PI3K) pathway and leads to the translocation of glucose transporter type 4 (GLUT4) to the cell surface, thus inducing glucose uptake. Alternatively, exercise stimulates glucose uptake in the muscles through GLUT4 in an insulin-independent manner. AMP-activated protein kinase (AMPK) is activated during exercise and promotes glucose uptake in the absence of insulin (Hayashi *et al*. 1997). Impairment of glucose uptake in the muscles is observed in pathological conditions such as obesity and diabetes. Contraction of the skeletal muscles stimulates GLUT4 translocation in an insulin-independent manner (Lund *et al*. 1995). Because glucose uptake during muscle contraction occurs in the absence of insulin, it is suggested that insulin-independent pathways may be involved in this event. However, the exact mechanisms underlying GLUT4 translocation through the sarcolemma during muscle contraction are unknown.

Isoeugenol (4-propenyl-2-methoxyphenol) is a naturally occurring *o*-methoxyphenol and a clear to pale yellow oily liquid present in various foods and essential oils, especially clove oil and cinnamon (Choi *et al*. 2007, Salanti *et al*. 2010). It is commonly used as a flavoring agent in baked foods, sweets, beverages, and frozen dairy products. Furthermore, it is widely used in cosmetics, dentistry, and traditional medicine. Because of the widespread use of these products, the potential toxicity of isoeugenol has been studied both *in vivo* and *in vitro* (George *et al*. 2001). Findings of several extensive studies indicate that isoeugenol is generally safe when used as a flavoring agent. Isoeugenol exerts anti-inflammatory effects, inhibits lipid peroxidation, and induces the detoxification of phase II enzymes (Choi *et al*. 2007). In addition, isoeugenol modulates several immune responses, including inflammation (Rajakumar & Rao 1993). In metabolism, eugenol has been suggested as a promising therapeutic agent to prevent type 2 diabetes, after the demonstration that the compound effectively ameliorates hyperglycemia through inhibition of hepatic gluconeogenesis (Jeong *et al*. 2014). As eugenol is an analogue of isoeugenol, thus isoeugenol could be another molecular target for metabolic disease. However, no detailed information has been reported yet.

The AMPK complex is an evolutionarily conserved sensor of cellular energy status (Hardie 2004, Kahn *et al*. 2005). Once activated, AMPK switches on ATP-generating pathways and switches off ATP-consuming pathways. AMPK is activated by multiple AMPK regulatory pathways (Fryer *et al*. 2002, Hawley *et al*. 2002). The molecular mechanisms underlying AMPK activation are yet to be thoroughly elucidated. However, AMPK activation requires the phosphorylation of a catalytic α subunit at Thr-172 in its activation loop (Crute *et al*. 1998, Stein *et al*. 2000). Both liver kinase B1 (LKB1) and calcium/calmodulin-dependent protein kinase kinase (CaMKK) are involved in AMPK activation (Hawley *et al*. 2003, Woods *et al*. 2003, Hong *et al*. 2005, Hurley *et al*. 2005). AMPK plays a pivotal role in glucose uptake in an insulin-independent manner. *In vitro* studies have shown that isolated muscles exposed to 5-aminoimidazole-4-carboxamide-1-β-ribofuranoside (AICAR) show increased glucose uptake in the absence of insulin (Hayashi *et al*. 1998, Bergeron *et al*. 1999, Koistinen *et al*. 2003). Therefore, it can be suggested that isoeugenol-induced AMPK activation is a promising target for regulating glucose uptake in an insulin-independent manner. However, not many candidates have been identified as successful antidiabetic agents thus far.

In this study, we investigated the effects of isoeugenol on AMPK phosphorylation in the muscles to precisely characterize its metabolic effects. We observed that isoeugenol increased AMPK phosphorylation and glucose uptake through p38 MAPK and AS 160 pathways.

## Materials and methods

### Reagents

STO-609 (CaMKK inhibitor) and isoeugenol were purchased from Sigma Chemical Company. AICAR was purchased from Toronto Research Chemical Incorporation (Toronto, ON, Canada). SB203580 (p38MAPK inhibitor) and BAPTA-AM (a cell-permeant chelator) were purchased from Abcam (Cambridge, MA, USA). Polyclonal antibodies against phosphorylated AMPKα, phosphorylated ACC, phosphorylated p38 MAPK, and phosphorylated AS160 and antibodies against AMPKα, ACC, p38 MAPK, AS160, and β-actin were purchased from Millipore (Billerica, MA, USA). Compound C (AMPK inhibitor) was provided by Merck (Rahway, NJ, USA). Hybond ECL nitrocellulose membranes were obtained from GE Healthcare (Little Chalfont, Buckinghamshire, UK).

### Cell culture

Mouse C2C12 myoblasts and rat L6 myoblasts were maintained in DMEM supplemented with 10% heat-inactivated FBS and 1% antibiotics (100 U/ml penicillin and 100 μg/ml streptomycin) at 37 °C in a humidified atmosphere of 5% CO_2_. Rat L6 myoblasts were seeded in 12-well plates at a density of 2×10^4^ cells/ml for differentiation into myotubes that were used in glucose uptake studies. After 24 h (at >80% confluence), the medium was replaced by DMEM containing 2% (v/v) FBS. Thereafter, the medium was replaced after 2, 4, and 6 days of culture. Experiments were initiated after 7 days when myotube differentiation was complete.

### Western blot analysis

The cells were grown in six-well plates. After achieving 60–70% confluence, the cells were serum starved for 24 h before treatment with selected agents at 37 °C. The cells were then treated with 30 μM isoeugenol for 3 h. After the treatment, the medium was aspirated. The cells were washed twice with ice-cold PBS and were lysed in 100 μl lysis buffer (0.5% deoxycholate, 0.1% SDS, 1% Nonidet P-40, 150 mM NaCl, and 50 mM Tris–HCl (pH 8.0)) containing proteinase inhibitors (0.5 μM aprotinin, 1 μM phenylmethylsulphonyl fluoride, and 1 μM leupeptin; Sigma). The supernatants were sonicated briefly, heated for 5 min at 95 °C, centrifuged for 5 min, separated on SDS-polyacrylamide gel (8–16%), and transferred onto PVDF membranes. The membranes were incubated overnight with primary antibodies at 4 °C, after which they were washed six times with Tris-buffered saline containing 0.1% Tween-20. The membranes were then incubated with HRP-conjugated secondary antibodies for 1 h at room temperature. Anti-β-actin antibody was used to normalize protein loading. The blots were visualized using an ECL solution (GE Healthcare).

### Uptake of 2-deoxy-d(H^3^)-glucose

Glucose uptake was analyzed by measuring the uptake of 2-deoxy-d(H^3^)-glucose (2-DG) by differentiated L6 myotubes. The cells were rinsed twice with warm PBS (37 °C) and were starved in serum-free DMEM for 3 h. After isoeugenol treatment, the cells were incubated in KRB (20 mM HEPES (pH 7.4), 130 mM NaCl, 1.4 mM KCl, 1 mM CaCl_2_, 1.2 mM MgSO_4_, and 1.2 mM KH_2_PO_4_) containing 0.5 μCi 2-DG at 37 °C for 15 min. The reaction was terminated by placing the plates on ice and by washing the cells twice with ice-cold PBS. The cells were then lysed in 0.5 N NaOH, and 400 μl of cell lysate was mixed with 3.5 ml scintillation cocktail. Radioactivity was measured by scintillation counting.

### RT-PCR

First-strand cDNA was synthesized using 1 μg of total RNA from C2C12 cells at 55 °C for 20 min by using Thermoscript II One-Step RT-PCR Kit (Life Technologies). The cDNA was amplified using GeneAmp PCR System 9700 (Applied Biosystems), followed by heating to 94 °C for 5 min to inactivate the reverse transcriptase. PCR was performed using 34 cycles of denaturation at 94 °C for 30 s, annealing at 55 °C for 30 s, and amplification at 72 °C for 60 s, followed by final elongation at 72 °C for 10 min. The number of PCR cycles was optimized to ensure that the amplification was performed in an exponential phase. Next, 10 μl of the PCR products were analyzed by performing agarose gel electrophoresis. The bands obtained were stained with ethidium bromide and were visualized under u.v. light. Band intensities were quantified using UVP BioDoc-It imaging system (Upland, CA, USA). The PCR was performed using the following primers: GLUT4 sense (5′-TTG GAG AGA GAG CGT CCA AT-3′) and GLUT4 antisense (5′-CTC AAA GAA GGC CAC AAA GC-3′) and β-actin sense (5′-CAG GAG GAG CAA TGA TCT TGA-3′) and β-actin antisense (5′-ACT ACC TCA TGA AGA TCC TCA-3′). Each experiment was repeated three times.

### Measurement of intracellular calcium

Intracellular calcium concentration was measured by detecting the fluorescence of cells treated with a calcium-sensitive indicator fluo-3 AM. Fluorescence was detected using a confocal microscope (Zeiss LSM 700; Zeiss, Deutschland, Oberkochen, Germany). The cells were treated with 5 μM fluo-3 AM in a regular culture medium for 45 min at room temperature. After washing with the medium, the cells were incubated in the absence of fluo-3 AM for 15 min to completely de-esterify the dye. Culture plates were placed on a temperature-controlled microscope stage and were observed under 20× objective. Signal was detected at an excitation and emission wavelength of 488 nm.

### Silencing of genes encoding AMPKα2, AMPKα1, and AS160

The cells were seeded in six-well plates and were cultured to 70% confluence for 24 h. The cells were then transiently transfected with siRNAs against genes encoding AMPKα2, AMPKα1, and AS160 (L-040809, L-091373, and L-040174, Dharmacon, GE Healthcare) by using Lipofectamine 2000 (Invitrogen, Life Technologies), according to the manufacturer's protocol. For transfection, 5 μl of the siRNAs and 5 μl of Lipofectamine 2000 were diluted using 95 μl of reduced serum medium (Opti-MEM; Invitrogen, Life Technologies) and were mixed. The mixture was incubated for 30 min at room temperature and was added dropwise to each culture well containing 800 μl of Opti-MEM (final siRNA concentration, 100 nM). The medium was replaced with a fresh complete medium after 4 h of transfection.

### Myc-GLUT4 translocation assay

Cell surface expression of Myc-GLUT4 was quantified by performing an antibody-coupled colorimetric absorbance assay, as described previously (Wijesekara *et al*. 2006). After DHA stimulation, L6 myotubes that stably expressed Myc-GLUT4 were incubated with polyclonal anti-Myc antibody (1:1000 dilution) for 60 min, fixed with 4% paraformaldehyde in PBS for 10 min, and incubated with HRP-conjugated goat anti-rabbit antibody (1:1000 dilution) for 1 h. The cells were then washed six times with PBS and were incubated in 1 ml *o*-phenylenediamine (0.4 mg/ml) for 30 min. Absorbance of the supernatant was measured at 492 nm.

### Preparation of primary myoblasts

Primary myoblasts were isolated from the forelimbs and hindlimbs of 3–4 5-day-old littermates (Bois & Grosveld 2003). The muscles were dissected and minced, were disaggregated enzymatically in 4 ml PBS containing 1.5 U/ml dispase II and 1.4 U/ml collagenase D (Roche), and were triturated with a 10-ml pipette every 5 min for 20 min at 37 °C. The cells were filtered through a 70-μm mesh (BD Bioscience, CA, USA) and were centrifuged at 1000× ***g*** for 5 min. The cell pellet was dissociated in 10 ml F10 medium (Invitrogen, Life Technologies) supplemented with 10 ng/ml basic fibroblast growth factor (PeproTech, Rocky Hill, NJ, USA) and 10% cosmic calf serum (referred to as growth medium 1; GE Healthcare). Finally, the cells were pre-plated twice on non-collagen coated plates for 1 h to deplete fibroblasts that generally adhere faster than myoblasts. For differentiation, the primary myoblasts obtained were cultured to 75% confluence in DMEM containing antibiotics and 5% horse serum (Invitrogen, Life Technologies).

### Data analysis

One-way ANOVA, Holm–Sidak comparisons, and Fisher's *post hoc* test were used to compare the potency of glucose uptake. The difference between mean values was considered statistically significant when *P* was <0.05.

## Results

### Isoeugenol stimulates glucose uptake through AMPK phosphorylation in C2C12 cells

**Figure 1:**
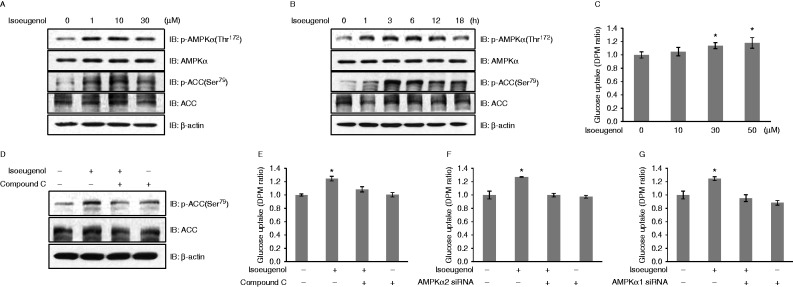
Isoeugenol stimulates glucose uptake by phosphorylating AMPK in C2C12 cells. (A) C2C12 cells were stimulated with isoeugenol for 1 h, and cell lysates were analyzed by performing western blotting with antibodies against phosphorylated AMPKα and phosphorylated ACC. Non-phosphorylated AMPKα and ACC were used as controls. β-actin served as protein loading control. (B) The cells were stimulated with 10 μM isoeugenol for the indicated time, and cell lysates were analyzed by performing western blotting with antibodies against phosphorylated AMPKα and phosphorylated ACC. Non-phosphorylated AMPKα and ACC were used as controls. β-actin served as protein loading control. (C) L6 myoblasts were differentiated for 7 days and were stimulated with various doses of isoeugenol for 1 h, and 2-DG uptake was assayed; *P<0.05 compared with control cells. (D) C2C12 cells were pretreated with compound C. The cells were then treated with 10 μM isoeugenol for 1 h, and cell lysates were analyzed by performing western blotting with an antibody against phosphorylated ACC. Non-phosphorylated ACC was used as a control. β-actin served as protein loading control. (E) L6 myoblasts were differentiated and were stimulated with 10 μM isoeugenol for 1 h in the presence of compound C, and 2-DG uptake was assayed; *P<0.05 compared with isoeugenol-treated cells. (F) Differentiated L6 myotubes were transfected with siRNA against the gene encoding AMPKα2 and then stimulated with isoeugenol for 1 h, and 2-DG uptake was assayed; *P<0.05 compared with control cells. (G) Differentiated L6 myotubes were transfected with siRNA against the gene encoding AMPKα1 and then stimulated with isoeugenol for 1 h, and 2-DG uptake was assayed; *P<0.05 compared with control cells.

To determine whether isoeugenol exerted metabolic effects in C2C12 cells, we evaluated its effects on AMPK, the key regulator of glucose uptake. Administration of isoeugenol induced a dose- and time-dependent increase in AMPK phosphorylation in C2C12 cells (Fig. 1A and B). The concentration of isoeugenol at 10 μM increased AMPK phosphorylation to the maximum. The degree of AMPK phosphorylation increased to maximum at 30 min after isoeugenol treatment. Phosphorylation of ACC, a downstream target of AMPK, also increased after isoeugenol administration, which was consistent with the increase in AMPK phosphorylation. Next, we characterized the functional importance of AMPK activation. Glucose uptake is a good parameter to test the significance of AMPK activation. Among skeletal muscle cells, differentiated L6 myotubes showed higher glucose uptake than C2C12 cells, suggesting that L6 myotubes were the most promising model for investigating glucose uptake (Sarabia *et al*. 1990). Accordingly, the effect of isoeugenol on glucose uptake was investigated in differentiated L6 myotubes. It was observed that isoeugenol increased glucose uptake in a dose-dependent manner (Fig. 1C). Pretreatment with 5 μM compound C blocked ACC phosphorylation and glucose uptake induced by isoeugenol, suggesting that AMPK played a role in isoeugenol-induced glucose uptake (Fig. 1D and E). Knockdown of the gene encoding AMPKα2 decreased isoeugenol-induced glucose uptake (Fig. 1F). Knockdown of AMPKα1 also decreased isoeugenol-induced glucose uptake (Fig. 1G). These results indicated that both AMPKα2 and AMPKα1 were involved in isoeugenol-induced glucose uptake.

### Intracellular calcium mediates isoeugenol-induced AMPK phosphorylation and glucose uptake

**Figure 2:**
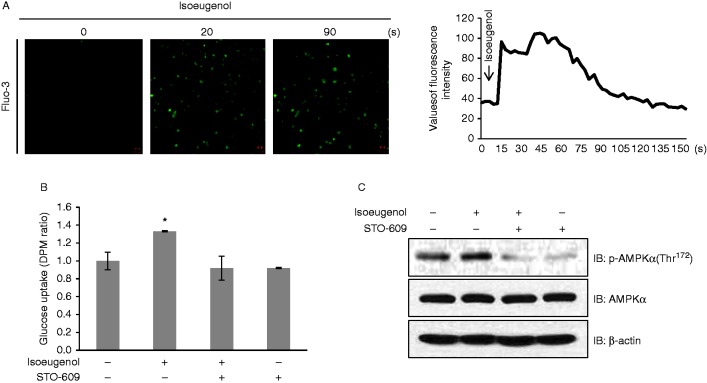
Intracellular calcium concentration regulates isoeugenol-induced AMPK phosphorylation and glucose uptake. (A) C2C12 cells were pretreated with fluo-3 AM for 30 min and then with 10 μM isoeugenol, and green fluorescence was detected using a confocal microscope. (B) L6 myoblasts were differentiated for 7 days and were pretreated with STO-609 (5 μM) and isoeugenol (10 μM) for 1 h. Uptake of 2-DG was assayed; *P<0.05 compared with control cells. (C) C2C12 cells were pretreated with STO-609 for 30 min and then with 10 μM isoeugenol for 1 h. Cell lysates were analyzed by performing western blotting with antibodies against phosphorylated AMPKα. Non-phosphorylated AMPKα was used as controls. β-actin served as protein loading control.

Increase in intracellular calcium concentration activates AMPK (Hardie *et al*. 2003). Therefore, we hypothesized that calcium acted upstream of AMPK. To test this hypothesis, we measured intracellular calcium concentration by using fluo-3 AM. Fluorescence intensity indicated the degree of calcium concentration. Isoeugenol increased the intensity of green fluorescence (Fig. 2A), indicating an increase in intracellular calcium concentration. This result indicated that CaMKK was an upstream component of the AMPK pathway. To confirm this, C2C12 cells were pretreated with STO-609, a CaMKK inhibitor, before treatment with isoeugenol. STO-609 blocked isoeugenol-induced glucose uptake (Fig. 2B) and AMPK phosphorylation (Fig. 2C), thus confirming that isoeugenol increased glucose uptake through the calcium-mediated CaMKK–AMPK pathway.

### PKCα is involved in isoeugenol-induced glucose uptake

**Figure 3:**
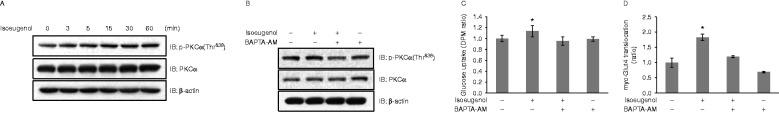
PKCα is involved in isoeugenol-induced glucose uptake. (A) C2C12 cells were stimulated with 10 μM isoeugenol for the indicated times, and cell lysates were analyzed by performing western blotting with an antibody against phosphorylated PKCα. Blotting with antibodies against non-phosphorylated PKCα and β-actin were used as a control. (B) C2C12 cells were pretreated with BAPTA-AM (25 μM) for 30 min and then with 10 μM isoeugenol. Cell lysates were analyzed by performing western blotting with an antibody against phosphorylated PKCα. Blotting with antibodies against non-phosphorylated PKCα and β-actin was used as a control. (C) Differentiated L6 myotubes were pretreated with BAPTA-AM for 30 min and then with isoeugenol for 1 h. Uptake of 2-DG was assayed; *P<0.05 compared with control cells. (D) L6 myoblasts that stably expressed Myc-GLUT4 were differentiated for 7 days. The cells were pretreated with BAPTA-AM for 30 min and were incubated with isoeugenol for 1 h. Cell surface expression of Myc-GLUT4 was detected by performing antibody-coupled colorimetric absorbance assay; *P<0.05 compared with control cells.

PKCα is activated by calcium (Matta & Mobasheri 2014). This led us to test the involvement of PKCα in isoeugenol-induced AMPK phosphorylation. Because PKCα is regulated by calcium, we hypothesized that isoeugenol stimulated AMPKα signaling through PKCα. Isoeugenol increased phosphorylation of PKCα in a time-dependent manner (Fig. 3A). BAPTA-AM, a calcium chelator, suppressed isoeugenol-induced phosphorylation of PKCα (Fig. 3B). Moreover, BAPTA-AM blocked isoeugenol-induced glucose uptake (Fig. 3C) and GLUT4 translocation (Fig. 3D). These results indicated that isoeugenol induced glucose uptake through the PKCα pathway.

### Isoeugenol activates the p38MAPK pathway through AMPK

**Figure 4:**
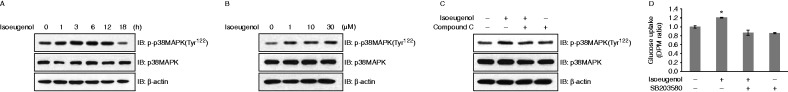
Isoeugenol activates the p38MAPK pathway in an AMPK-dependent manner. (A) C2C12 cells were stimulated with 10 μM isoeugenol for the indicated times. Cell lysates were analyzed by performing western blotting with an antibody against phosphorylated p38MAPK antibody. Antibody against non-phosphorylated p38MAPK was used as a control. β-actin served as protein loading control. (B) C2C12 cells were stimulated dose dependently with isoeugenol for 1 h. Cell lysates were analyzed by performing western blotting with an antibody against phosphorylated p38MAPK. Blotting with antibody against non-phosphorylated p38 MAPK was used as control. β-actin served as protein loading control. (C) C2C12 cells were pretreated with 5 μM compound C for 30 min and were treated with 10 μM isoeugenol. Cell lysates were analyzed by performing western blotting with an antibody against phosphorylated p38MAPK. Blotting with antibody against non-phosphorylated p38 MAPK was used as control. β-actin served as protein loading control. (D) Differentiated L6 myotubes were pretreated with SB203580 for 30 min and were treated with isoeugenol for 1 h. Uptake of 2-DG was assayed; *P<0.05 compared with control cells.

P38MAPK plays an important role in glucose uptake (Niu *et al*. 2003, Cheng *et al*. 2006). To understand the signaling pathways involved in isoeugenol-induced glucose uptake, we investigated the effects of isoeugenol on p38MAPK. Isoeugenol (10 μM) increased p38MAPK phosphorylation in a time- and dose-dependent manner (Fig. 4A and B). Pretreatment with 5 μM compound C blocked p38MAPK phosphorylation (Fig. 4C). SB203580, a p38MAPK inhibitor, suppressed isoeugenol-induced glucose uptake, suggesting that p38 MAPK played an important role in isoeugenol-induced glucose uptake (Fig. 4D). Together, these results indicated that p38MAPK functioned downstream of AMPK in the isoeugenol-induced pathway.

### Isoeugenol stimulates GLUT4 translocation in an AMPK-dependent manner

**Figure 5:**
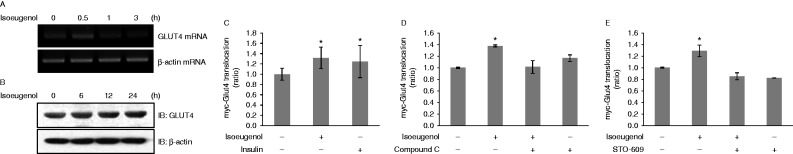
Isoeugenol stimulates GLUT4 translocation in an AMPK-dependent manner. (A) Total mRNA was extracted from isoeugenol-treated C2C12 cells. RT-PCR was performed using GLUT4-specific primers. The PCR products were separated on 1% agarose gels and were visualized under u.v. light. β-actin was used as a positive control. (B) The cells were stimulated with 10 μM isoeugenol for the indicated times. Cell lysates were analyzed by performing western blotting with an antibody against GLUT4. Blotting with an antibody β-actin served as control. (C) L6 myoblasts that stably expressed Myc-GLUT4 were differentiated for 7 days. The cells were then treated with isoeugenol for 1 h and with insulin for 15 min. Cell surface expression of Myc-GLUT4 was detected by performing antibody-coupled colorimetric absorbance assay; *P<0.05 compared with control cells. (D) The differentiated cells were pretreated with 5 μM compound C for 30 min and were treated with isoeugenol for 1 h. Cell surface expression of Myc-GLUT4 was detected by performing antibody-coupled colorimetric absorbance assay; *P<0.05 compared with control cells. (E) The differentiated cells were pretreated with 5 μM STO-609 for 30 min and were treated with isoeugenol for 1 h. Cell surface expression of Myc-GLUT4 was detected by performing antibody-coupled colorimetric absorbance assay; *P<0.05 compared with control cells.

GLUT4 is the main protein involved in glucose uptake in the skeletal muscles (Zhan *et al*. 2011). Effect of isoeugenol on GLUT4 expression was evaluated to determine the mechanisms underlying isoeugenol-induced glucose uptake. Isoeugenol increased GLUT4 mRNA and protein levels in C2C12 cells (Fig. 5A and B). Levels of Myc-GLUT4 on the plasma membrane increased after isoeugenol treatment, indicating that isoeugenol stimulated GLUT4 translocation from the cytosol to the plasma membrane (Fig. 5C). Insulin was used as a positive control. An increase in the levels of Myc-GLUT4 on the plasma membrane was not apparent in cells pretreated with compound C (Fig. 5D) and STO-609 (Fig. 5E), indicating that AMPK and calcium were involved in isoeugenol-induced GLUT4 translocation. These results suggested that isoeugenol regulated glucose uptake by stimulating GLUT4 translocation.

### Isoeugenol increases AS160 phosphorylation

**Figure 6:**
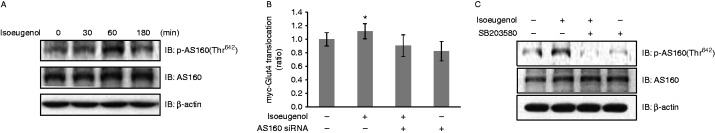
Isoeugenol increases AS160 phosphorylation. (A) C2C12 cells were stimulated with 10 μM isoeugenol for the indicated times. Cell lysates were analyzed by performing western blotting with an antibody against phosphorylated AS160. Blotting with antibodies against AS160 and β-actin served as control. (B) C2C12 cells were pretreated with 5 μM SB203580 for 30 min and were treated with 10 μM isoeugenol. Cell lysates were analyzed by performing western blotting with an antibody against phosphorylated AS160. Blotting with antibodies against non-phosphorylated AS160 and β-actin served as control. (C) L6 myoblasts that stably expressed Myc-GLUT4 were differentiated for 7 days. The cells were then transfected with siRNA (100 nM) against the gene encoding AS160 for 2 days and were incubated with isoeugenol for 1 h. Cell surface expression of Myc-GLUT4 was detected by performing antibody-coupled colorimetric absorbance assay; *P<0.05 compared with control cells.

AS160 controls GLUT4 translocation (Kramer *et al*. 2006). We hypothesized that isoeugenol controlled glucose regulation through AS160. To determine the mechanism underlying isoeugenol-induced glucose uptake, we examined the effect of isoeugenol on AS160 phosphorylation. Isoeugenol increased AS160 phosphorylation in a time-dependent manner (Fig. 6A). Pretreatment with SB203580 blocked isoeugenol-induced AS160 phosphorylation (Fig. 6B), indicating that p38 MAPK acted upstream of AS160. Silencing of the gene encoding AS160 blocked isoeugenol-induced GLUT4 translocation (Fig. 6C). These results indicated that isoeugenol induced GLUT4 translocation through the AMPK–AS160 pathway.

### Isoeugenol increases AMPK phosphorylation and stimulates glucose uptake in primary cultured myoblasts

**Figure 7:**
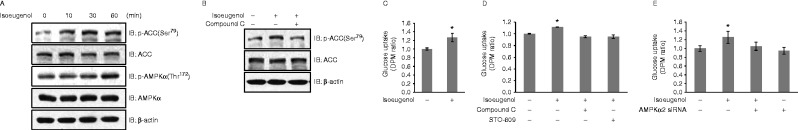
Isoeugenol activates AMPK and stimulates glucose uptake in primary cultured myoblasts. (A) Primary cultured myoblasts were stimulated with 10 μM isoeugenol for the indicated times. Cell lysates were analyzed by performing western blotting with antibodies against phosphorylated ACC and phosphorylated AMPKα. Blotting with antibodies against non-phosphorylated ACC, AMPKα, and β-actin served as control. (B) Primary cultured myoblasts were stimulated with isoeugenol for 1 h in the presence of compound C. Cell lysates were analyzed by performing western blotting with an antibody against phosphorylated ACC. Blotting with antibody against non-phosphorylated ACC was used as control. (C) Primary myoblasts were differentiated for 3 days. The cells were then stimulated with isoeugenol and metformin for 1 h, and 2-DG uptake was assayed; *P<0.05 compared with control cells. (D) The differentiated primary myotubes were pretreated with 5 μM compound C and STO-609 for 30 min and were treated with isoeugenol. 2-DG uptake was assayed; *P<0.05 compared with control cells. (E) The differentiated primary myotubes were transfected with siRNA against the gene encoding AMPKα2 for 2 days. The cells were then treated with isoeugenol, and 2-DG uptake was assayed; *P<0.05 compared with control cells.

To obtain insights on the *in vivo* effects of isoeugenol, we examined its effect on primary cultured myoblasts. Isoeugenol increased AMPKα and ACC phosphorylation in a time-dependent manner (Fig. 7A). Isoeugenol-induced ACC phosphorylation was suppressed by compound C (Fig. 7B). Further, isoeugenol increased glucose uptake in primary myotubes (Fig. 7C). Inhibition of AMPK and CaMKK abrogated the increase in isoeugenol-induced glucose uptake (Fig. 7D). To confirm the role of AMPK, the cells were transfected with siRNA against the gene encoding AMPKα2. This inhibited the increase in isoeugenol-induced glucose uptake (Fig. 7E). These results indicated that isoeugenol induced glucose uptake through the AMPK pathway in primary cultured myoblasts.

## Discussion

The principal finding of our study was that isoeugenol, a structural analog of curcumin, stimulated glucose uptake in skeletal muscle. This finding suggests that the hypoglycemic effects of curcumin can be attributed to metabolic effects similar to those exerted by isoeugenol in skeletal muscle. The glucose-lowering effect of isoeugenol was probably exerted through AMPK activation in skeletal muscle.

The hypoglycemic role of curcumin has been reported in a streptozotocin-induced diabetic animal model (Nishiyama *et al*. 2005). However, despite its clinical potential, curcumin has not yet been used as a therapeutic agent because of its poor absorption. The chemical structure of curcumin plays a critical role in its biological activity. Therefore, structural modifications such as use of analogs may enhance its solubility and bioavailability. In this study, we observed that isoeugenol increased AMPK phosphorylation in skeletal muscle. However, because we did not compare the metabolic role of isoeugenol in skeletal muscle, we cannot rule out the possibility that it is more effective than curcumin in regulating glucose. Collectively, these findings suggest that a novel structural motif exists in curcumin structural analogs and that isoeugenol represents this primary motif that affects glucose metabolism.

One study compared the antitumor activity of curcumin and isoeugenol (Fujisawa *et al*. 2004). This study showed that curcumin exerted better cytotoxic effects against cancer cells than isoeugenol. Because only a few studies have compared the effects of curcumin and isoeugenol on glucose regulation, we could not conclude whether curcumin or isoeugenol was a more promising candidate for developing antidiabetic drugs. Curcuminoids undergo successive reduction to its metabolites in the liver during phase I metabolism and are extensively conjugated with glucuronic acid during phase II metabolism. Approximately 75% of the administrated dose of curcumin is excreted unchanged in feces (Wahlström and Blennow 1978). In isolated hepatocytes, 90% of administered curcumin is metabolized within 30 min, suggesting that it is difficult to achieve a substantial concentration of curcumin. This result indicates that the *in vivo* instability of curcuminoids should be considered while evaluating their clinical usefulness. Another way to increase clinical utility is to develop structural analogs of isoeugenol that may alter its pharmacokinetics to make it more easily absorbable in the intestine or more readily metabolizable to a more stable form.

It was demonstrated that intracellular calcium signalling is associated with skeletal muscle atrophy (Zhou *et al*. 2010, Mirza & Tisdale, 2012). In this study, isoeugenol increased the intracellular calcium of skeletal C2C12 cells. Our study also showed that there was no stimulatory effect on glucose uptake or Glut4 translocation in the presence of STO-609, CaMKK inhibitor, indicating that skeletal muscle function, such as glucose uptake, was associated with isoeugenol-mediated calcium signaling. In addition, it is suggested that NF-κB-dependent inducible nitric oxide synthase (iNOS) is involved in skeletal muscle myotrophy (Altamirano *et al*. 2012). To gain further insight into the role of isoeugenol-mediated calcium release, in this study we examined the effect of calcium chelation of isoeugenol-mediated calcium on iNOS induction. There was no effect on iNOS, a muscle atrophy gene, indicating that muscle atrophy was not associated with isoeugenol-mediated calcium signaling. It is also known that autophagy is related to skeletal muscle functions, such as apoptosis inhibition (McMillan & Quadrilatero 2014) and muscle plasticity (Sanchez *et al*. 2014). Exercise-induced autophagy is known to be required for muscle glucose homeostasis (He *et al*. 2012). In this study, there was no effect of isoeugenol on autophagy genes (data not shown), indicating that autophagy was not associated with isoeugenol-mediated function.

Curcumin is considered as a potential candidate for the development of a treatment for metabolic diseases, such as diabetes. In this study, the EC_50_ of isoeugenol for glucose uptake was less than that of curcumin. This fact indicated that the potency of isoeugenol in stimulating glucose uptake was not less than curcumin. It was demonstrated that curcumin can ameliorate skeletal muscle atrophy in diabetic mice (Ono *et al*. 2015). Our previous study demonstrated that curcumin stimulated glucose uptake in an AMPK dependant manner (Kim *et al*. 2010), but curcumin had no effect on intracellular calcium concentration. However, isoeugenol stimulated glucose uptake through AMPK in a calcium dependent manner. In addition, curcumin ameliorated muscle atrophy, but isoeugenol did not. Combined with the fact that both agents have a similar structure, this led us to speculate that both agents have different roles with respect to intracellular calcium.

To understand which effects could improve glucose uptake on skeletal muscle in diabetes, we investigated the effect of isoeugenol on muscle atrophy-related genes, such as iNOS. Isoeugenol did not affect iNOS expression level, suggesting that the stimulatory effect of isoeugenol on glucose uptake may be due to a direct effect on GLUT4 trafficking-related molecules, such as AS160, rather than due to effect on muscle atrophy-related phenomenon. These findings provide a better understanding of the hypoglycemic functions of isoeugenol in skeletal muscle cells and suggest that isoeugenol is a promising agent for treating diabetes. In the future, more extensive studies should be performed on the relationship between the structure and functions of isoeugenol.

## Author contribution statement

All authors made contribution to the conception and design of the experiments. K N and J O L performed the research and developed methods. H S K designed the research. All authors were involved in drafting and all approved the manuscript.
